# Development of a 5As-based technology-assisted weight management intervention for veterans in primary care

**DOI:** 10.1186/s12913-018-2834-2

**Published:** 2018-01-29

**Authors:** Katrina F. Mateo, Natalie B. Berner, Natalie L. Ricci, Pich Seekaew, Sandeep Sikerwar, Craig Tenner, Joanna Dognin, Scott E. Sherman, Adina Kalet, Melanie Jay

**Affiliations:** 1 VA NY Harbor Healthcare System, Manhattan Campus 423 East 23rd Street, New York, NY 10010 USA; 2 New York University School of Medicine, 550 1st Avenue, New York, NY 10016 USA

**Keywords:** Obesity, Primary care, Intervention development, Patient centered medical home, VETERANS

## Abstract

**Background:**

Obesity is a worldwide epidemic, and its prevalence is higher among Veterans in the United States. Based on our prior research, primary care teams at a Veterans Affairs (VA) hospital do not feel well-equipped to deliver effective weight management counseling and often lack sufficient time. Further, effective and intensive lifestyle-based weight management programs (e.g. VA MOVE! program) are underutilized despite implementation of systematic screening and referral at all VA sites. The 5As behavior change model (Assess, Advise, Agree, Assist, Arrange) is endorsed by the United States Preventive Service Task Force for use in counseling patients about weight management in primary care and reimbursed by Medicare. In this paper, we describe the iterative development of a technology-assisted intervention designed to provide primary care-based 5As counseling within Patient-Centered Medical Homes without overburdening providers/healthcare teams.

**Methods:**

Thematic analyses of prior formative work (focus groups with patients [*n* = 54] and key informant interviews with staff [*n* = 25]) helped to create a technology-assisted, health coaching intervention called Goals for Eating and Moving (GEM). To further develop the intervention, we then conducted two rounds of testing with previous formative study participants (n = 5 for Round 1, n = 5 for Round 2). Each session included usability testing of prototypes of the online GEM tool, pilot testing of 5As counseling by a Health Coach, and a post-session open-ended interview.

**Results:**

Three main themes emerged from usability data analyses: participants’ emotional responses, tool language, and health literacy. Findings from both rounds of usability testing, pilot testing, as well as the open-ended interview data, were used to finalize protocols for the full intervention in the clinic setting to be conducted with Version 3 of the GEM tool.

**Conclusions:**

The use of qualitative research methods and user-centered design approaches enabled timely detection of salient issues to make iterative improvements to the intervention. Future studies will determine whether this intervention can increase enrollment in intensive weight management programs and promote clinically meaningful weight loss in both Veterans and in other patient populations and health systems.

## Background

The prevalence of obesity in the United States (US) has increased over the past several decades [1], and recent data show that over one-third of adults in the US have obesity and over two-thirds have a body mass index (BMI) in the overweight range. Among Veterans, the prevalence of obesity is slightly higher [2, 3]. The United States Preventive Services Task Force (USPTF) recommends that all patients be screened for obesity and offered intensive lifestyle counseling since this can lead to modest weight loss and decreased risk of chronic disease [4]. Thus, Veterans Affairs (VA) Medical Centers require that all Veterans in primary care are screened for obesity by their Patient Aligned Care Teams (PACT) and referred to the VA MOVE! weight management program.

PACTs were originally launched in 2010 to implement a Patient-Centered Medical Home (PCMH) model of primary care at VA hospitals [5, 6]. PCMHs aim to improve primary care delivery through care that is patient-centered, comprehensive, increases access to services, and improves quality and safety [7, 8]. PACTs promote a whole person, team-based approach to coordinate Veterans’ medical, behavioral, and psychosocial healthcare needs [5]. The MOVE! weight management program follows evidence-based obesity treatment guidelines and has a comprehensive, multidisciplinary approach to weight management with group or individual meetings, often facilitated by dieticians or behavioral psychologists [9–13]. The MOVE! program is effective [14–16], but only 8% of eligible Veterans attend at least 1 MOVE! visit [17]. This underutilization of an existing intensive weight management program presents the need for other treatment options for those who do not attend, as well as increased support to promote attendance to MOVE!. Since Veterans receiving care at VAs see a primary care provider (PCP) an average of 3.6 times per year [18], primary care has the potential to be a valuable setting for both promoting healthy weight management behaviors and encouraging patients to attend MOVE!.

The 5As model has shown increasing promise in delivering a range of behavioral interventions within the primary care setting and is endorsed by the USPTF [19]. Medicare also reimburses practices for performing 5As counseling [20], and the Canadian Obesity Network endorses use of a similar model [21]. This model provides a framework for clinicians to effectively counsel patients through the completion of 5 specific groups of tasks (see Table 1). These include encouraging clinicians to “assess” current beliefs, behavior, and knowledge, “advise” lifestyle changes based on specific information about health risks, “agree” on collaborative goals based on the patient’s interest and confidence, “assist” the patient to achieve these goals by identifying barriers and creating strategies, and “arrange” for a specific follow-up plan to track progress [19, 22, 23]. Goal-setting, corresponding to the “agree” component of the 5As, is supported by the Theory of Planned Behavior [24] and other behavior change theories as a way to foster behavior change [25, 26]. Goal setting has been shown to promote both diet and physical activity changes and lead to weight loss [27, 28].

**Table 1:** 5As Framework for Obesity Counseling and the how intervention components align with tasks

5As Framework for Obesity Counseling		Intervention Components			
5As	Tasks	Online Tool	PACT Members	Health Coach	Telephone Coaching
ASSESS	Risk, Stage of Change, Current Behaviors	✓			
ADVISE	Weight loss, Behavior Change	✓			
AGREE	Collaboratively set goals	✓	✓	✓	✓
ASSIST	Address barriers, Motivational Interviewing		✓	✓	✓
ARRANGE	Follow-up, Referrals		✓	✓	✓

*Abbreviation*: *PACT* patient aligned care teams

Several studies have demonstrated the feasibility and practicality of adapting the 5As model to deliver obesity interventions within the primary care setting [21, 29–33]. Another study demonstrated that training physicians in 5As-based counseling resulted in modest patient weight loss at 12 months [34]. However, a major barrier to implementing the 5As in clinical practice is that PCPs and other healthcare team members often fail to counsel patients for many reasons including competing demands on time [35]. One way to address barriers to providing 5As-based obesity care within primary care settings is to use interactive behavior change technologies to assist with several tasks in the 5As model. These interactive technologies have the ability to assess behaviors/barriers, generate tailored advice, facilitate goal setting, and promote behavior change. Indeed, a recent systematic review demonstrated that technology-assisted weight loss interventions in the primary care setting helped patients to achieve weight loss compared to usual care [36]. In addition, they can also help to address the well-documented time constraints of clinicians. Thus, interventions using technology-assisted goal setting have the potential to overcome barriers and facilitate 5As-based weight management counseling.

Based on our prior research and experience training providers to deliver 5As [30, 32, 37], as well as other studies showing that time and competing demands could limit implementation and sustainability of 5As counseling [21, 29, 31], we sought to develop an intervention that would facilitate 5As counseling without overburdening providers/healthcare teams and also increase enrollment in MOVE!. In this paper, we describe how we used qualitative research methods and user-centered design approaches to develop and pilot test a 5As-based, technology-assisted weight management intervention to improve obesity care for Veterans within primary care.

## Methods

The intervention, rooted in the 5As framework, is based on the Theory of Planned Behavior where intention to perform a new behavior predicts behavior change [24]. Studies show that interventions that produce greater changes in intention are more likely to produce behavior change [38]. It is also based on current goal setting theory where achieving small, doable goals increases a patient’s self-efficacy to set and achieve more goals [39, 40]. Our approach to intervention development was based on the ORBIT model, a systematic framework for guiding efforts to translate basic behavioral science findings into behavioral treatments for preventing and treating chronic illness, which recommends iterative design with frequent pilot testing [41]. We iteratively developed intervention components with a user-centered design approach and piloted them between July 2014 and July 2015. We utilized a multidisciplinary research team of VA clinicians, health science and public health students/professionals, software developers, dietitians, physical activity specialists, as well as extended advisors and consultants from the MOVE! weight management program and the VA National Center for Health Promotion and Disease Prevention. Here, we summarize this process in two phases: the “Development Phase” and the “Testing Phase” [see Table 2].

**Table 2:** Overview of study methods

Development Phase • Thematic analysis of formative research data (focus groups with Veterans and key informant interviews with Veterans Affairs hospital staff) to identify intervention components • Iterative development of online weight management counseling tool ○ Paper prototypes to outline overall front-end tool design and a layout, described back-end functionality logic, store/track tool content (ex. questions and advice) ○ Prototypes developed and improved through usability testing (see Testing Phase below)
Testing Phase • Round 1 sessions: December 2014 • Round 2 sessions: May 2015 • Sessions included: ○ Usability testing of either Prototype 1 (Round 1) or Prototype 2 (Round 2) of online tool ○ Pilot Testing of 5As counseling with an expert clinician as health coach (Round 1) or a trained research team member as health coach (Round 2) ○ Open-ended exit interviews with participants

The Development Phase consisted of 1) a thematic analysis of previous formative research (Veteran focus groups and key informant interviews with PACT staff) [42, 43] to determine intervention components, and 2) iterative development of an online weight management tool that facilitates goal setting (later named “Goals for Eating and Moving” or “GEM tool”). The Testing Phase consisted of two rounds of concurrent usability testing [44] of the GEM tool with Veterans, and pilot testing of other components of the intervention, including health coaching materials. Round 1 of testing occurred in December 2014 and Round 2 in May 2015. The protocols for both phases were approved by the local VA Institutional Review Board and participating Veterans consented to be part of all study procedures. Additionally, all sessions were audio-recorded, with focus groups, key informant interviews, and usability testing sessions transcribed professionally.

### Development phase

#### Thematic analysis of formative research to identify intervention components

To inform development of intervention components, we conducted a thematic analysis of existing and previously coded transcripts of qualitative data from two formative research studies: six focus groups with Veteran patients (2 female, 4 male, *n* = 54) (occurred September 2013) and twenty-five key informant interviews with VA clinical staff including PCPs, RNs, and MOVE! staff (occurred March-September 2013) [42, 43]. The average age of our Veteran patients was 58 year old and 74% had completed college or graduate school. Notably, our sample population of Veteran patients was over-representative of women, Hispanic individuals, and African Americans compared to 2015 national Veteran demographic characteristics (63% male vs. 92% nationally, 13% Hispanic vs. 6% nationally, and 46% African American vs. 11% nationally) [45]. Among our sample population of VA clinical staff, 21% were male, 46% identified as non-White, and the average age was 45 years old. Additional details of participant characteristics for the focus groups and key informant interviews are described elsewhere [42, 43].

A subset of the original transcript codes common to both data sets (“goal-setting,” “technology,” and “proposed intervention”) were analyzed by two members of the research team (SS and KFM) in order to identify major themes related to intervention development. These themes were discussed and clarified with the larger team and PI to guide development of intervention components and processes (see Table 3). Additional details regarding the qualitative methods used in the original coding and analysis of these data sets are described elsewhere [42, 43].

**Table 3:** Major themes and supporting evidence from formative research analysis, and corresponding intervention components

Theme	Evidence	Intervention Components
Collaborative goal setting	• Veterans and VA staff felt positively about using goal setting for healthy behavior change • VA staff felt patients often set unrealistic goals, but VA staff burden increased when they had to work with patients to scale goals down	• *Online tool* creates initial goals • *Health coach* helps refine into SMART goals • *PACT members* endorse goals and provide motivational interviewing if needed
Accountability and Feedback	• Veterans wanted someone to hold them accountable to their goals and receive advice from their primary care team • VA staff faced time constraints when discussing goal setting during the PC visit • VA staff described lack of effective or standardized way to record patient goals and communicate them to their PACT	• *Health coach* primarily delivers intervention and refines goals with Veterans • *PACT members* use an EMR research note and automatic reminder to discuss/update goals during next visit • *Telephone coaching* allows Health Coach to regularly follow up Veterans, document progress, and adjust goals
Assistance with Technology	• Some Veterans were familiar with various technology platforms and used tools to research health information and/or facilitate healthy behaviors, while others felt uncomfortable using technology without guidance • Veterans and VA staff agreed that a knowledgeable individual should be available to assist patients in using technology	• *Online tool* has built-in instruction slide • *Health coach* is present to answer questions and/or guide Veteran while using online tool
Difficulties with Transportation	• Veterans described barriers (ex. physical disabilities and/or financial issues) to traveling to the VA for scheduled primary care appointments or MOVE! sessions	• *Telephone coaching* allows Health Coach to meet with Veterans via telephone at convenient times for the patient

*Abbreviations*: *EMR* electronic medical record, *PC* primary care, *VA* veterans affairs

#### Overview of key intervention components

Based on this secondary qualitative data analysis described above, we designed the intervention components to facilitate 5As counseling (see Table 1). We conceived that a tablet-delivered online tool would assess health behaviors/barriers (“assess”), provide tailored advice (“advise”), and help patients set initial goals (“agree”). Members of the PACT healthcare team would then discuss goals further by addressing barriers (“assist”) and provide follow-up to more intensive support (“arrange”). Due to extensive discussion with PACT members around time constraints for weight management counseling during the primary care visit, we decided to add a trained Health Coach to the team. The term “health coach” is usually defined as someone who helps individuals adopt and maintain healthy behaviors [46]. In this intervention, the role of the Health Coach is to provide support for the online tool, refine goals (“agree”), discuss barriers (“assist”), and follow up through telephone coaching (“agree”). For the intervention, we designed the Health Coach role to be filled by a non-clinically trained person (often a graduate student or recent college graduate) whose role would be to help patients adopts and maintain healthy behaviors. The addition of this Health Coach to PACTs would allow the healthcare team to focus their limited time on brief (< 5 min) counseling to address barriers and endorse goals. The telephone coaching would allow for more counseling continuity. A clinical electronic medical record (EMR) reminder would prompt the PACTs to review Health Coach notes and facilitate documentation of counseling.

#### Iterative development of online weight management counseling tool

We iteratively developed the online weight management counseling tool based on the original MOVE!23, now called the MOVE!11. The MOVE!11 is an expert system software program with an 11-item online questionnaire (the original MOVE!23 was a 23-item online questionnaire) that was developed as an intake tool for new patients in the MOVE! weight management program [47]. It evaluates current eating and physical activity habits, as well as barriers to weight management, and then provides tailored advice and links to patient education handouts. In a previous study with Latina women, we found that the MOVE!23 did not adequately support goal setting and participants had difficulty using a mouse/trackpad on desktop/laptop computers to complete the MOVE!23 and preferred touchscreens [48]. In addition, other studies have discussed the ability of tablets to improve technological self-efficacy among older adults and assessed the acceptability of touch-screen technology among low-income primary care patients [49, 50]. We used this data and consultation with the VA’s National Center of Prevention to inform the development of an initial prototype of the Goals for Eating and Moving (GEM) tool to support goal setting and 5As counseling on tablet computers. We sought to design a tool that would: (1) assess lifestyle and weight management behaviors, (2) provide tailored advice and patient education materials, (3) guide patients to select initial goals around weight loss, diet, and physical activity based, and (4) facilitate counseling by a Health Coach.

We first formulated initial paper prototypes using Microsoft PowerPoint and Excel (July-September 2014) to outline overall front-end tool layout, describe important business logic, and store/track tool content (i.e. question/answer/linked advice). Then, with the guidance of developers in PHP, Javascript, and HTML, we built the online tool using a LAMP Web stack (short for Linux, Apache, MySQL, and PHP), an open-source web development platform that uses Linux as the operating system, Apache as the Web server, MySQL as the relational database management system (RDMS) and PHP as the object-oriented scripting language to return advice to the user. Several working prototypes optimized for use on iPad tablets were developed, tested, and improved upon (October 2014-July 2015) both internally (among the research team) and externally (with Veteran participants). The research team and additional software programmers and designers met weekly to review and discuss iterative improvements to the system using all paper and working prototypes.

The first prototype of the GEM tool was designed at a 5th grade literacy level with simple navigation and optimized for delivery via an iPad for portability within clinic settings. It was designed such that no personally identifiable data is collected, and any information entered by the patient (e.g. lifestyle behaviors/barriers, goals, preferred resources) is saved and linked only by a randomly generated identification number. Additionally, information entered is only accessible by the research team and shared with the patient’s PACT (clinical team) via the EMR. With this Version 1 of the GEM tool, we developed and tested a unique goal setting process:

1. With guidance from the Health Coach, the tool asks a series of 16 questions about the patient’s lifestyle behaviors/barriers and then provides tailored weight loss and behavior change advice. For each piece of advice, the online tool guides the patient to report the perceived importance of the advice on a 10-point Likert Scale.
2. The tool generates a ranked list of potential goals based on the advice and user importance rating.
3. The patient choses a weight loss goal, nutrition goals, and a physical activity goal from the list with the option to write in other potential goals.
4. The patient then receives a personalized binder of tailored materials generated by the tool and assembled by the Health Coach to facilitate the creation of SMART goals and further Health Coach counseling.

### Testing phase

Veterans who previously participated in focus groups from our formative research studies [43] were invited back to participate in a one-on-one Testing Phase session with research team members trained in usability/interviewing protocols and note-taking practices, as well as one specifically trained as a Health Coach). These one-on-one sessions took place in two rounds (Round 1 in December 2014 and Round 2 in May 2015) and included: in-depth usability testing of a GEM tool prototype, pilot testing of 5As counseling assisted by tool-generated tailored materials, a brief open-ended interview to receive feedback about the entire session, and pre/post-surveys. Each component of this phase is described in more detail below. Veterans were each given a $40 cash voucher for participating.

#### Usability testing

We aimed to recruit 5-6 Veteran patients per two rounds of in-depth usability testing, adhering to recommendations in the literature regarding appropriate sample size [51–53]. With one research team member trained in usability methodology, Veterans used a prototype of the GEM tool on an iPad tablet while following a “Think-Aloud” protocol [44, 54, 55], a cognitive interviewing technique and common usability evaluation method which allows researchers to gain insight into participants cognitive strategies and processing during problem-solving activities [44, 54, 56, 57]. Immediate verbalizations while interacting with the tool describe participants’ cognitive responses to a situation more accurately than retrospective interviews [44, 54, 56, 58, 59]. Participants were asked questions such as “what do you think of this page?” or “how do you feel about these answer choices?” to guide them through the Think-Aloud process and obtain feedback on the tool. Interactions were audio-recorded while another research team member trained in note-taking practices took field notes on interactions with each screen of the tool, specifically related to aspects of the tool that hindered or facilitated usability to guide rapid and iterative development of the tool.

#### Pilot testing of 5As counseling

**Fig. 1:**
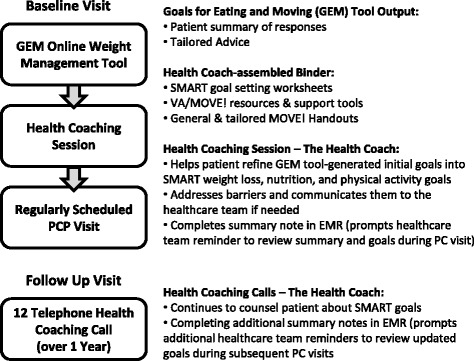
5As Intervention Design (Abbreviations: EMR = electronic medical record, PC = primary care)

The 5As counseling session, which occurred immediately following a usability testing session of the online tool component, was facilitated by a research team member taking the role of a Health Coach who followed a structured health coaching guide to complete the “Agree”, “Assist”, and “Arrange” intervention components. We created the health coaching session structure to incorporate GEM tool-generated materials (see Fig. 1). 5As counseling during Round 1 of testing was conducted by the Principal Investigator (MJ), an attending physician at the Manhattan VA, while 5As counseling during Round 2 was conducted by a trained member of the research team who had no formal clinical training (KFM). This trained Health Coach received 10 h of training in motivational interviewing, role-playing, the 5As Model, SMART (Specific, Measurable, Attainable, Relevant, and Timely) goal setting [60], and referring to the MOVE! program.

#### Open-ended interview

After each 5As counseling session, Veterans were asked structured, open-ended questions by a separate member of the research team member trained in interviewing about the their overall experience using the GEM Tool and participating in a brief 5As counseling session with a Health Coach, as well as other proposed components and protocols for the larger intervention (see Table 4). These questions were asked without the Health Coach in the room in order to encourage both positive and constructive feedback. In between testing rounds, audio recordings of the health coaching sessions and responses to the open-ended questions were reviewed and discussed by the research team to make improvements to the health coaching guide, tool-generated materials, and implementation protocols.

**Table 4:** Open-ended interview questions

General	What did you like best about the experience?
	What did you like least about the experience?
	Do you have any other comments about anything today?
GEM Tool	How likely are you to recommend this to another veteran?
	If you did the questionnaire and health coaching right before seeing your nurse or doctor, • Who on the team would you want to discuss your goals with? • What would be the best way to discuss these goals with the team?
	If you could change anything about the GEM questionnaire, • What would you change? • What would you leave the same?
	The GEM questionnaire was designed to get information from you in order to provide advice and help you set goals. • How useful was the advice for setting goals? • What could make it more useful?
	How can the GEM questionnaire help you better set goals?
	If you were going to see your doctor, • Where would you like complete the questionnaire beforehand (physical location)? • How would you feel about taking it at home? • How would you feel about taking it at the clinic?
Health Coaching Session: General	Overall, how was the experience talking about your weight, lifestyle, and goals with the health coach? • What could make the health coaching experience more useful?
	What is your understanding of what a SMART goal is?
	How was your experience writing down your goals on the worksheet? • What could make it better?
	What would be the best way for us or healthcare providers to contact you and check in with you about your progress starting and maintaining these goals?
	If a health coach, not a doctor, scheduled phone calls to check about your progress, • How would you feel about that? • How often would you like these phone calls to be? • Are there any other ways you would like someone to follow up with you on your goals?
	Do you have any other feedback about the health coaching session?
Health Coaching Session: Tailored Materials	The personal report summarized your answers to the questionnaire and wrote out all the tailored advice. • What do you think of the personal report you received? • What do you think of the binder and the handouts you received? • What do you think you will do with them when you get home?

### Data analysis

Transcripts from the Think-Aloud portion of usability testing were analyzed using a three-tier coding system created with guidance from the usability framework provided by the International Organization for Standardization (ISO) [59]. This approach including the use of an a priori codebook, the process of segmenting and coding the text, the negotiating of coding conflicts, the use a summative content analysis approach to incorporate code frequency as to identify trends in data, and the inclusion of all coded statements in synthesizing themes has been used in our previous research [48]. Briefly, first tier codes described the interactions between four domains of usability – user, tool, task, and context (e.g., user-tool, tool-task), second tier codes categorized the type of interaction between the domains (enables, impedes, or wants), and third tier codes described the main topic of the interaction that was vocalized by the participant (e.g., experience eating, question language). Transcripts were coded separately by two individual coders who met frequently to resolve disagreements. The final coded segments were analyzed for code frequencies using R statistical software package [61], and the research team met frequently to discuss data trends and synthesize recurring themes and factors. Audio recordings from open-ended interviews were reviewed by a member of the research team, who took detailed notes on relevant feedback needed to make improvements to the tool and intervention components, as well as inform the implementation of the intervention in the primary care setting.

## Results

### Testing phase

Ten unique Veteran patients participated in a Testing Phase session, which included usability testing of a prototype of the tool, a 5As counseling session, and a brief open-ended interview (five in Round 1 and five in Round 2). Participants were mostly male (60%), African American (60%), and had an average age of 62.23 years (see Table 5). Usability sessions lasted on average 108 min (range 77-144, SD 19.53), with the Think-Aloud portion lasting an average of 63.5 min (range 33-90, SD 16.37), 5As counseling sessions lasting an average of 27.7 min(range 20-36, SD 5.42), and open-ended interviews lasting an average of 16.8 min (range 9-22, SD 3.82).

**Table 5:** Demographics of Usability Testing Sample (*n* = 10)

	n (%)
Race^a^	
White	3 (30)
Black or African	6 (60)
American	0 (0)
Asian	0 (0)
American Indian	1 (10)
Other	0 (0)
Ethnicity	
Hispanic	1 (10)
Not Hispanic	0 (0)
Gender	
Male	6 (60)
Female	4 (40)
Age (years)	
Mean	61.3
Median	62
BMI	
Mean	31.10
SD	3.36

^a^Participants could select more than one race

Three main themes emerged after analyzing data from Rounds 1 and 2 of usability testing (see Table 6). Briefly, the first theme was that the tool language elicited an emotional response from users. Emotional responses were both positive and negative, with generally more negative comments occurring in Round 1. The second theme was that users sought clarification when the purpose of a question was unclear. Veteran participants would seek support from their Health Coach while progressing through the tool, or received clarification provided through extra information via hyperlink “pop-ups” within the tool itself. A third theme was that answer options and advice needed to be clear at the appropriate health literacy level. Veterans who were unfamiliar with medical terminology struggled because it was not explained or defined. Open-ended interviews confirmed many of the findings from the usability testing and observations from the 5As counseling sessions. Below, we describe findings in more detail.

**Table 6:** Usability Testing Themes (with domain interaction code), Factors, and Example Veteran Quotes

Theme 1	Theme 2	Theme 3
Tool Language Elicits Emotional Response (USER-TOOL)	Users Seek Clarification When Question Purpose is Unclear (USER-CONTEXT/USER-TOOL)	Answer options & advice must be clear & health literate (USER-TOOL)
Round 1 Example Quotes		
MOD: So how did you feel when you were reading this paragraph…that’s telling you to lose weight? US-08: Well I’m getting scolded. US-01: It’s…telling you you’re fat and these people, they’re here because they know they’re fat…And they want to lose weight. MOD: Okay. US-01: Could you reword it? Try not to gain weight, I would recommend that you try not to, not that you do not. It’s a little nicer, I think.	US-06: What do you think may get in the way to change your eating habits, check all that apply…Used to eating a certain way, I don’t understand the question. MOD: What do you think it might mean? US-08: Just to eating a certain way like (long pause) I don’t know. US-08: Make my goals into smart goals, I’m not sure I know what that means. MOD: Okay, all right so it’s unclear at this page what you’re supposed to do? US-08: Right.	US-02: Now I got a question with the eating disorder…Binge eating, anorexia or bulimia. Okay maybe I binge eat…but I don’t consider it something out of control because I don’t do it all the time, I may do it once a month. MOD: How would we make that more clear…? US-02: I would say maybe if it’s something they do more than maybe twice a month or something, you know, put a number on it, you know... US-05: So it’s asking me for my ID number right, your ID number or is this saying there is an ID number up here right?... So where would I have this ID number…would it be on my military card or what? MOD: So it’s unclear kind of what the ID number is for. US-05: Yes.
Round 2 Examples Quotes		
MOD: So it says at the top “lost over 45 pounds.” What do you think about having these show up? US-11: That’s good, it shows they’re motivated and they’re doing something positive for themselves. US-13: I like the fact that…you have the motivation part, that’s great keep it up. I like that I really like that.	US-14: Okay, why is this (clicks on red underline). MOD: What do you think about that information? US-14:: I love that …it’s telling me … something I know but you see I always go for the bigger numbers because then it will motivate me even more. But I like the fact it gives you smaller increments, you know, it’s telling you to choose smaller increments and why. MOD: Do you know why that is red and underlined? US-09: Yes it would tell me in detail.	MOD: How do you feel about that advice? US-04: Great. No she was very good, very good and if you’re in doubt you just tell them go ask my coach or the healthcare team where I can go get help. MOD: So what do you think about this slide? US-04: Well in case I didn’t know, this will help me to know…It’s informative. It will give me information I need to at least start.

*Abbreviations*: *MOD* moderator; US-## = Respondent ID)

#### Findings from usability testing – Round 1

In Round 1, users found that the tool did not entirely facilitate task completion. Regarding the first theme, Veterans were uncomfortable with some of the language in the tool, describing it as “rude” or “hurtful” especially when the tool told them “your height and weight puts you at a very high risk for many health conditions…we advise you to lose weight.” The majority of participants wanted gentler, more supportive language, while a minority of them appreciated the terse nature of the tool’s advice, stating that it felt motivational and deserved. In addition, word choice or long questions were cited as points of confusion for participants and prevented them from being able to answer without assistance. Finally, medical terminology caused confusion; participants were unfamiliar with certain medical concepts used in the tool and were unsure of what to do with the information given. Participants also cited issues with tool-specific words and phrasing. This was echoed during open-ended interviews where several Veterans expressed frustration when questions were difficult to understand. While there were some functionality issues (i.e., sensitivity/ touch response) and visual preferences (i.e. larger font letters and button sizes), Veterans were more concerned with understanding how to complete the tool. Based on these Round 1 sessions, we made changes to tool content (i.e. included more collaborative and supportive language, added definitions) and made overall improvements to the user interface and design with assistance from a designer (see Table 7). Importantly, we reorganized the flow of questions and advice in order to connect related content, as well as separated the goal-setting process into multiple steps to make it easier to follow and reduce the amount of information presented to Veterans at one time.

**Table 7:** Changes to GEM Tool between Version 1 and Version 2 (after Usability Testing – Round 1)

• Tutorial question added
• Status bar replaced with time approximation
• Question sequence was altered to group goals by type (weight loss, nutrition, physical activity)
• Mini-summaries were added throughout the tool
• Background colors changed from tan to blue and white
• VA logo added
• Terminology definitions added via embedded hyperlinks
• Tool language softened and clarified
• Expanded Veteran resources list
• Categorized advice added after each question
• Tutorial question adapted for relevance

#### Findings from usability testing – Round 2

During the Round 2 sessions, Veterans responded positively to the iterative changes made to the tool (see Table 7). Veterans were able to more independently use the tool and found it aesthetically pleasing. Additionally, the participants reported that the tool was easy to navigate, and they understood what they were being asked. Answer choices were also well received, as they thought the tool was inclusive of most possible choices. However, a few Veterans wished for more “none” options, since despite the 5-10 choices for some questions, none of them applied to them personally. Veterans appreciated that terminology was defined, clear, and had examples to better facilitate understanding. Functionality of the tool remained problematic during some sessions, including insufficient sensitivity of the touchscreen. While occasionally frustrating for Veterans, this did not prevent task completion. Changes following Round 2 focused on simplifying the wording of questions, removing/consolidating answer choices that caused confusion, and rearranging the physical activity section of the tool to better facilitate goal setting.

#### Findings from open-ended feedback: GEM tool

From open-ended interview data, Veterans overall found the online tool useful for creating personalized goals and appreciated motivating language, and the tailoring of information based on their responses. Most negative feedback related to technical difficulties in using the tool. One Veteran mentioned wanting the tool to be more tailored to individuals with diabetes or other conditions like heart disease or health situations like post-surgery or post-pregnancy. All Veterans said they would recommend the online tool to another Veteran after the technical issues were resolved, but noted that other Veterans would be willing to use it only if they were motivated to take serious steps to lose weight and improve their health. Responses differed when asked about where Veterans would prefer to complete the tool. Some preferred to complete it at home because they would not feel rushed to complete it, while other Veterans preferred to complete it while in the clinic waiting area before their appointment or more generally in the hospital environment where there would be fewer distractions. Whether in the clinic area or at home, Veterans wanted a private space to complete the online tool with the support from someone to provide assistance if needed.

#### Findings from open-ended feedback: Health coaching sessions

Overall, almost all of the Veterans had a positive experience with health coaching and found the session informative and personalized: “[the] individualized session it’s more personal … you don’t have to feel rushed or you don’t have to feel like you’re taking away from somebody else” (Round 1, US-14). Veterans found the counseling helpful particularly to explain concepts that they did not understand, connect them to other resources, and also receive encouragement. One patient noted about the Health Coach: “The phone numbers she gave me for the cooking classes…and the making an appointment with a nutritionist. No one ever told me about that (Round 1, US-11).” However, several preferred that the Health Coach give more direction and preferred having the Health Coach write down their goals. One patient pointed out he wanted specific information about what “should” be done and preferred that a professional write down exactly what he needs to do to achieve his goals (Round 1, US-05). Another Veteran felt that the Health Coach did not have enough specialized knowledge to answer specific question about diet or nutrition, and would have preferred having other specialists at the session (Round 2, US-11).

Most Veterans had a very strong positive response to the personalized binder materials presented and discussed during the health coach counseling session. “That’s why I liked it…the individualization…this is like mine, nobody else has one like this, you know, this is one of a kind it’s not like where you’ve gone and just made up a whole bunch of booklets and we all get them. This, I feel like this has been customized personally for me (Round 1, US-02).” Many expressed that they would go home and read through the material in more detail and at their own pace: “It’s not a dust collector…it’s usable (Round 2, US-02).” Many found the SMART goal setting worksheets helpful to specifically put onto paper the goals discussed with their Health Coach. One Veteran preferred discussing goals verbally but not necessarily writing them down (Round 2, US-09).

When asked with whom on their PACT team they would prefer to discuss their goals, almost all Veterans cited their doctor, though many felt that the nurse most likely had more time to look up their medical records, listen, and have a discussion**.** Generally, Veterans wanted to discuss their goals with someone who had the time and interest to have a personalized conversation. All Veterans preferred that the Health Coach and/or healthcare provider followed up on their goals by telephone. Veterans expressed wanting to receive these calls anywhere from once a week to every two weeks to calling every couple of months so that they could get feedback regarding their progress. Regardless of how often they preferred the Health Coach or PACT member call, most of the patients highlighted the importance of having a “real” person they could talk to and preferably the same person to see growth or changes over time.

### Description of proposed intervention design

Findings from both rounds of usability testing the health coaching sessions, as well as the open-ended interview data were used to finalize protocols for the full intervention in the clinic setting, to be conducted with Prototype 3 of the GEM tool. The full intervention integrates with regularly scheduled PCP visits by requiring eligible patients (Veteran patients age 21-70 years old with a BMI greater than 30 kg/m^2^ or greater than 25 kg/m^2^ with co-morbidities) to arrive approximately 45 min prior to their appointment. These Veteran patients will be identified pro-actively by automatically generated lists of eligible patients with upcoming appointments. The Health Coach gives a brief introduction to the GEM tool and allows the patient to work through the tool. The Health Coach is present to answer any questions, but only if prompted by the patient. When the GEM tool is complete, the Health Coach assembles the tailored components of the binder.

The health coaching session begins when the binder is complete, and follows a protocol that includes the creation of at least one nutrition and one physical activity SMART goal, using handouts in the binder especially designed to facilitate making SMART goals. The Health Coach walks the patient through each component of the binder and provides brief introductions to Pedometer use as well as the use of a food journal (copies enclosed in the binder).

Upon completion of Health Coaching sessions, patients either continue straight to their PCP appointment, or the Health Coach completes a warm handoff to the MOVE! program staff for either MOVE! enrollment or meetings with staff dietitians. During the PCP appointment, the PCP receives an automatic prompt within the VA Computerized Patient Record System (CPRS) to complete the GEM Study “Reminder” and discuss goals with the patient. PCPs have the Health Coach note at their disposal for review during the appointment.

Follow up phone coaching sessions (with Health Coaches) are scheduled periodically for one year. In-person study visits occur at 3, 6, and 12 months. Phone coaching sessions, developed based on baseline coaching sessions and designed to check in on goals, occur every two weeks for the first 3 months, monthly for the second three months, and bimonthly for the last six months. Health Coaches write notes in CPRS following each phone coaching session, cosigned by the team dietitian, so that the whole PACT team can stay up to date on their patients’ progress, and are aware of any medical concerns. Health Coach training also includes recognition of any patient-related issues at these additional points of contact outside of the scheduled primary care visit that may require immediate PCP notification, as well as a process for communicating these issues to PACT staff.

This proposed intervention is designed to maximize integration within PACT, reduce the burden on the PCP, and increase enrollment in MOVE! (see Fig.1). The individual components were carefully and iteratively designed to maximize 5As weight management counseling and utilize multiple members of the healthcare team, while providing optimal goal-related support to the patient.

## Discussion

In this paper, we describe the development of a technology-assisted 5As weight management counseling intervention. Commonly described approaches to intervention development and testing, and especially ones that incorporate behavior change and/or technology, include: intervention mapping [62–64], adaptive intervention design [65], person-based or user-centered approach [66–69], requirement development approach [70], behavior change wheel approach [71], participatory approach, EVOLVE model, ORBIT model, and other variations of systematic and theory-based behavioral science and mixed-methods approaches [41, 72–77]. What is often reiterated by authors utilizing these intervention approaches, and particularly the ORBIT model is the importance of both systematic planning and iterative design, guided by a theoretical basis or framework for behavior change, needs assessments or formative studies, and then continuous and integrated user input/feedback to identify and prioritize key intervention components.

Thus, our approaches to the development of the GEM intervention are well-supported in the literature. We designed the GEM intervention to address a crucial need to improve primary care-based weight management counseling, increase attendance to intensive programs such as MOVE!, and facilitate weight loss in patients who do not attend. We chose to use the 5As behavior change model as it has already been shown to be effective in smoking cessation counseling and is now seen as a promising framework for obesity counseling within primary care [34, 78]. We also wanted to focus on goal-setting as the key approach to weight management as it is an integral part of the 5As framework as well as a key element in several behavior change theories [24]. We used rigorous formative methods, combining perspectives from both Veteran patients and PACT staff to identify, prioritize, and develop initial intervention components prior to usability testing [42, 43]. We also used rigorous user-centered approaches, conducting two rounds of usability testing and evaluation, pilot testing of health coaching protocols, and open-ended exit interviews to guide iterative development of intervention components and incorporate crucial user feedback throughout the testing phase. We believe that these methods will increase the likelihood that our intervention will facilitate seamless delivery of 5As counseling integrated within the PCMH model of primary care at VA hospitals.

The GEM intervention has evidence-based components that have shown to be efficacious in other primary-care based weight management interventions. For instance, there have been three Practice-based Opportunities for Weight Reduction (POWER) studies, funded by the NIHLBI, which represent the most comprehensive primary care-based weight management studies to date [79–81]. Together, the POWER studies showed that various primary care-based weight management interventions can produce clinically significant weight loss in primary care practice. The use of non-clinician support staff (e.g., health coaches, medical assistants, health educators) was an important component of all these interventions, and two incorporated technology. However, none of these studies were delivered within medical homes nor did they integrate with existing, intensive weight management programs at the respective sites. The GEM intervention is innovative because it leverages the PCMH model of primary care to improve implementation of the 5As framework through the use of technology-assisted, collaborative goal setting by medical home-based health coaches integrated within primary care teams.

## Limitations

Several methodological limitations should be acknowledged and addressed. For usability testing of the early prototypes of the GEM tool, different professionals (i.e. clinician vs. non-clinician) were involved in Round 1 and Round 2 of testing, which may have resulted in different outcomes related to health counseling. However, we purposely had a clinician (MJ, the PI) provide the health coaching in Round 1 to ensure that the health coaching protocol was relevant and feasible in a best-case scenario. We then had a non-clinician (KFM) trained in the protocols be the Health Coach in Round 2 in order to assess whether someone without formal clinical training could assist patients with using the tool and provide brief 5As counseling. Another limitation is that Veteran patients that previously participated in our formative research were invited back to participate in usability testing. Our intent in using this approach was to involve members of our target population in the prototype testing of our tool. Inviting back study participants from our formative research allowed us to highlight how their focus group feedback was used to inform design of our tool and intervention. Additionally, we recruited only 5 Veteran patients per round of usability testing. There is no universally accepted standard sample size for usability testing, but existing literature recommends considering quality of assessment over quantity, while keeping in mind operational limitations [51–53]. Our iterative approach used two rounds of in-depth testing with members from our target population, combined with internal testing and development with a multidisciplinary team of clinicians, dietitians, other health professionals, software developers, and MOVE! program staff. As part of an on-going pilot RCT, we are further testing the feasibility of using non-clinician health coaches with more extensive training, as well as the acceptability of the intervention among a larger sample size of Veteran patients that have not involved in our formative or development research.

Another important limitation is that practices that do not use a PCMH model could potentially find this intervention difficult to implement, especially if they lack additional resources and programs to support weight management. However, multi-professional, team-based care is highly valued and increasingly being adopted [82]. We also acknowledge that much of the formative and development work to design the GEM intervention was conducted at a single VA site, and thus may not be directly translate to other settings and populations. However, a strength of the Manhattan VA is that it is one of the most diverse VA hospitals within the Veterans Affairs Healthcare System; compared to both national and New York state Veteran demographics, our sample population was more inclusive and representative of minority groups, specifically female, African American, and Hispanic Veterans [45]. Importantly, we utilized a multidisciplinary research team to design and test the GEM tool and intervention. Thus, although the GEM intervention was developed at a single site, we believe that the diverse training and perspectives of our team were key to developing a complex intervention that is widely applicable and has the potential to be implemented and easily tailored to other VA sites and other healthcare settings more broadly [83, 84].

## Conclusion

We describe a systematic intervention development process that aligns with the ORBIT model of behavioral intervention development. The use of qualitative research methods and user-centered design approaches enabled us to quickly detect salient issues and make iterative changes to prototypes of the GEM tool, improve health coaching protocols, and strengthen the overall design, and will facilitate integration of the intervention into primary practice. We believe that through this rigorous process, the resulting intervention has a higher likelihood to improve delivery of weight-management care for patients within primary care settings. We are currently testing the proposed intervention within the clinic setting for feasibility and acceptability as part of a pilot randomized controlled trial (RCT). Future studies will test the efficacy of this intervention in other VA centers as well as public outpatient centers.

## Abbreviations

BMI: Body mass index
CPRS: Computerized patient record system (EMR system of VA hospitals)
EMR: Electronic medical record
GEM: Goals for eating and moving
PACT: Patient aligned care teams
PCMH: Patient-Centered Medical Home
PCP: Primary care provider
RCT: Randomized controlled trial
RN: Registered nurse
SMART: Specific, measurable, attainable, relevant timely
US: United States
USPTF: United States Preventive Services Task Force
VA: Veterans affairs
